# Tinea Versicolor in a Child

**Published:** 1882-07

**Authors:** 


### TINEA VERSICOLOR IN A CHILD.

According to Dr. L. D. Bulkley (*Archives of Dermatology*, July, 1881), it is doubtful if tinea versicolor ever appears before puberty. Duhring ("Diseases of the Skin") has never observed it in children. Malcolm Morris ("Manual of Skin Diseases") says it does not occur in childhood. Special interest therefore attaches to the case reported by Dr. Walter G. Smith (*Archives of Dermatology*, January, 1882). A girl, aged twelve, was admitted into the hospital, under his charge, for measles. When convalescing from the measles it was noticed that she was suffering from an extensive eruption of tinea versicolor. The disease was widely spread over the chest, and was plainly visible on the back. It consisted for the most part of small dark brown spots and patches with abundant branny desquamation, but was not itchy. The eruption was confluent over the sternal region. The disease had existed for a very long time.—*Chic. Med. Rev.*

